# Tier 1 evaluating the implementation of the Oliver McGowan Mandatory Training in learning disabilities and autism across interdisciplinarity health-related courses at Aston University

**DOI:** 10.3389/fmed.2026.1672094

**Published:** 2026-03-10

**Authors:** Amreen Bashir, Karan Singh Rana, Jayne Murphy, Mary Drozd

**Affiliations:** 1 Medicine Pharmacy and Biosciences, Aston University, Birmingham, United Kingdom; 2 Psychology Health and Clinical Science School, Aston University, Birmingham, United Kingdom

**Keywords:** autism and learning disabilities awareness, educating healthcare students, equality, diversity and inclusion, inclusive teaching and learning, Oliver McGowan Mandatory Training

## Abstract

**Introduction:**

The Oliver McGowan Mandatory Training (OMMT) on Learning Disability and Autism was developed to address recognized gaps in healthcare professionals' education and training. Under the Health and Care Act, 2022, healthcare providers are required to ensure staff receive role-appropriate training in autism and learning disabilities. In response, NHS England introduced OMMT as a standardized national programme to support the development of a skilled and inclusive healthcare workforce, aligned with the NHS Long Term Workforce Plan (2023). This study reports on the first pilot implementation of Tier 1 OMMT within a higher education institution, involving students enrolled in Nursing, Biomedical Science, Physician Associate, Optometry, and Pharmacy programmes at Aston University, UK.

**Materials and methods:**

The interdisciplinary Tier 1 training consisted of a 90-min e-learning module and a 1-h interactive webinar featuring experts by experience and facilitators. This study evaluated healthcare students' understanding pre- and post-training. Data on autism and learning disabilities awareness was collected via Jisc Online Surveys. Likert scale data were analyzed quantitatively, and free-text responses examined using thematic analysis to evaluate training effectiveness.

**Results:**

Post-OMMT training results showed significant improvements in students' confidence in verbal communication and using various methods to communicate with autistic individuals and those with learning disabilities (*p* < 0.0001). Students reported enhanced understanding and awareness of autism and mild, moderate, severe and profound learning disabilities (*p* < 0.0001). Thematic analysis highlighted those students valued learning from experts by experience. Post-training participants recognized the benefits of individualized healthcare, the need for reasonable adjustments, and the importance of multidisciplinary team approaches in providing equitable care for autistic people and people with learning/intellectual disabilities.

**Discussion:**

This interdisciplinary training enhanced healthcare students' understanding of autism and learning disabilities, equipping them with key skills for future NHS roles and supporting improved outcomes for neurodivergent populations. Embedding such training across all HEIs is essential to prepare professionals to not only treat, but also understand, respect, and advocate for autistic and learning-disabled individuals.

## Introduction

Autism and learning disabilities are lifelong neurodevelopmental conditions characterized by substantial heterogeneity in presentation, support needs, and lived experience. Autism is associated with differences in communication, social interaction, sensory processing, and behavior, with characteristics typically emerging in early childhood, although identification may occur at any stage of life (1). Learning disabilities may affect a person's ability to understand complex information, acquire new skills, and live independently, with impacts that range from mild to profound (2, 45). Both autism and learning disabilities exist along broad spectrums, and individuals' strengths, needs, and health experiences vary considerably.

Despite this diversity, autistic people and people with learning disabilities, experience consistently poorer health outcomes and inequitable access to healthcare compared with the general population. A substantial body of evidence documents barriers including communication difficulties, diagnostic overshadowing, lack of reasonable adjustments, limited continuity of care, fragmented services, and insufficient understanding among healthcare professionals. These barriers contribute to delayed diagnosis, inappropriate treatment, avoidable harm, and premature mortality (3, 52, 54).

In England, national reviews have repeatedly demonstrated the scale and persistence of these inequalities. Findings from mortality surveillance and review programmes indicate that a significant proportion of deaths among people with learning disabilities are avoidable and are associated with failures in healthcare delivery rather than clinical complexity alone (4). Recurrent themes include poor communication, failure to recognize deterioration, lack of personalized care, and limited engagement with families and carers who hold essential knowledge about individuals' needs.

The death of Oliver McGowan has become emblematic of these systemic failures. Oliver McGowan was an autistic person with a mild learning disability who experienced repeated hospital admissions related to seizures. Despite clear documentation of contraindications to antipsychotic medication and repeated warnings from his family, inappropriate prescribing occurred, leading to catastrophic health deterioration and death at the age of 18 (5). This case is not presented as an isolated tragedy, but rather as illustrative of widespread and preventable failures identified across national evidence, particularly the failure of healthcare professionals to listen to families and to adapt care to individual needs.

Access to healthcare for autistic people and people with learning disabilities is shaped by a range of legislative and policy frameworks in England (47), including the Autism Act (6), the Equality Act (7), the Health and Care Act (8), and Care Act (44). The Autism Act was the first disability-specific legislation in England and places a statutory duty on the government to produce and regularly update a national autism strategy aimed at improving outcomes across health, social care, and education [(6), c. 15]. It emphasizes the importance of staff training, improved access to services, and the development of autism-informed pathways to reduce inequalities in care. Subsequent autism strategies developed under the Act have repeatedly identified workforce knowledge and understanding as key barriers to effective implementation. These frameworks collectively aim to promote equity, inclusion, and reasonable adjustments and place a statutory requirement on health and care providers to ensure staff receive training in autism and learning disabilities appropriate to their roles. However, despite these legal protections, substantial gaps remain between policy intentions and everyday practice, resulting in continued disparities in healthcare access, safety, and outcomes.

Multiple investigations into health and care services have demonstrated that legal safeguards are not consistently upheld (45–47, 49, 53). National inquiries and inspections have identified patterns of institutional neglect, inappropriate use of restraint, prolonged inpatient stays, and failures in safeguarding autistic people and people with learning disabilities (9).These findings highlight systemic shortcomings in workforce knowledge, attitudes, and skills, rather than isolated service failures, and reinforce the need for meaningful educational interventions that translate legislation into practice.

One of the most persistent challenges underpinning these disparities is the preparedness of the healthcare workforce. Research consistently indicates that healthcare professionals report limited confidence and competence in caring for autistic people and people with learning disabilities, particularly in relation to communication, reasonable adjustments, and person-centered decision-making (10). These gaps are evident across professional groups and are often attributed to insufficient coverage of autism and learning disability education within undergraduate and pre-registration curricula.

Interprofessional education (IPE) has been widely promoted as a mechanism for addressing complex healthcare challenges that require coordinated, multidisciplinary responses. Evidence suggests that IPE enhances collaborative working, communication, role understanding, and shared decision-making, contributing to improved patient care outcomes (11–14). For autistic people and people with learning disabilities, whose care frequently involves multiple professionals across health and social care, interdisciplinary approaches are particularly relevant.

A growing body of literature evaluates educational interventions designed to improve healthcare students' and professionals' competence in supporting autistic people and people with learning disabilities. Internationally, programmes delivered to medical students and residents have demonstrated improvements in awareness, confidence, and ability to provide personalized care for individuals with intellectual and developmental disabilities (15). Within the UK, co-produced learning disability education initiatives have similarly shown positive impacts on undergraduate healthcare students' understanding, confidence, and preparedness (16). A recent scoping review of learning disability education for medical students identified a wide variety of interventions, including classroom-based activities, placements, and panel discussions, with substantial variation in content, delivery, and evaluation (17). Most interventions incorporated lived experience, improving engagement and skill development, but the majority were elective rather than mandatory. This optional status risks reinforcing the hidden curriculum, limiting exposure, and signaling that intellectual disabilities are less important. Methodological limitations, including non-validated surveys and inconsistent outcomes, make effectiveness difficult to assess. These findings highlight the need for standardized, mandatory, co-produced educational programmes that ensure all students gain the knowledge and skills to provide equitable care.

Studies focusing specifically on autism education have reported improvements in communication skills, understanding of reasonable adjustments, and readiness for practice following dedicated training (18–20). Collectively, this literature demonstrates that a range of educational approaches can be effective in improving knowledge, confidence, and attitudes toward autism and learning disabilities. The Oliver McGowan Mandatory Training (OMMT) represents one such intervention, with a distinctive emphasis on national standardization and co-production with experts by experience. Although Tier 1 is designed as a foundational programme for the wider workforce and general public, it has been adopted within healthcare education settings to support early professional learning. Its alignment with statutory training requirements provides a clear rationale for evaluating its implementation and perceived impact within higher education contexts, particularly as a preparatory intervention prior to progression to role-specific Tier 2 training (51).

This study is grounded in Transformative Learning Theory (56), which posits that learning involves critically reflecting on prior assumptions, integrating new knowledge, and applying it in practice. By incorporating structured experiential learning, interprofessional collaboration, and reflection, the OMMT aims to foster genuine shifts in students' perspectives on inclusive care. Complementing this, Critical Disability Theory (21) and Autism Studies provide a lens emphasizing lived experience, affirmative approaches, and social justice, ensuring that the training prioritizes the voices and rights of autistic people and people with learning disabilities. This theoretical grounding positions the evaluation to assess not only knowledge acquisition but also attitudinal and behavioral transformation in future healthcare professionals.

Building on the gaps identified in healthcare education and grounded in the theoretical perspectives of Transformative Learning Theory and Critical Disability Theory, the Tier 1 OMMT was introduced in higher education to equip healthcare students with foundational knowledge and practical skills to support autistic people and people with learning disabilities. The training combines online e-learning modules with interactive sessions co-produced and co-delivered by experts with lived experience, ensuring that students engage with authentic perspectives and real-world challenges. By fostering reflection, critical thinking, and application in practice, the OMMT aligns with Transformative Learning Theory, enabling students to critically examine assumptions, develop inclusive attitudes, and adapt behaviors in future clinical settings. Its incorporation into undergraduate healthcare curricula addresses a key gap in mandatory education, ensuring that students are prepared to provide equitable, person-centered care from the outset of their professional development.

Aston University participated as a stakeholder in the West Midlands pilot rollout of Tier 1 OMMT within higher education institutions. Multiprofessional teams across the College of Health and Life Sciences collaborated with regional NHS partners and Integrated Care Boards to explore how the training could be delivered across interdisciplinary healthcare programmes and aligned with workforce development priorities.

This study aims to evaluate the implementation of Tier 1 OMMT within undergraduate health-related courses at Aston University, including Nursing, Biomedical Science, Physician Associate, Optometry, and Pharmacy programmes. Specifically, it examines the impact of the training on students' understanding of autism and learning disabilities, their confidence in communication and applying reasonable adjustments, and their preparedness for future roles within the NHS. By situating the evaluation within Transformative Learning Theory and disability-affirmative frameworks, this study seeks to determine whether the training fosters shifts in knowledge, attitudes, and behaviors. Evaluating OMMT in a higher education context allows the study to contribute empirical evidence on how mandatory, co-produced education can bridge the gap between legislation, professional education, and clinical practice, supporting safer, more inclusive, and person-centered healthcare for autistic people and people with learning disabilities.

## Materials and methods

The Oliver McGowan Mandatory Training is a standardized programme developed and evaluated by NHS England, and it has been designed to be delivered as mandatory training for all healthcare professionals. To evaluate its effectiveness in enhancing healthcare students' understanding of autism and learning disabilities, Biomedical Science and Nursing researchers at Aston University developed a bespoke survey specifically aligned with the Tier 1 OMMT learning outcomes. While validated instruments such as the Attitudes Toward Intellectual Disability Scale (22) and the Autism Stigma and Knowledge Questionnaire (23) exist, these tools primarily measure general attitudes or stigma rather than the targeted competencies addressed by OMMT. A bespoke survey allowed us to directly assess changes in students' perceived knowledge, confidence, and preparedness in key areas emphasized by the training, including communication strategies, implementation of reasonable adjustments, and delivery of person-centered care. This tailored approach provided a precise, context-specific evaluation of the training's impact within higher education, capturing outcomes directly relevant to a mandatory national programme and its role in preparing future healthcare professionals.

Aston University was one of the pilot sites selected to implement the training across Higher Education Institutions (HEIs) delivering health-related courses. Participating programmes include Biomedical Science, Nursing, Pharmacy, Physician Associates and Optometry. Completion of Tier 1 OMMT was mandatory within participating health-related programmes, in line with NHS England pilot implementation. However, completion of the research surveys was voluntary and not linked to assessment or progression.

### Ethical approval

The pre- and post-surveys were disseminated using the JISC Online Surveys platform. The Participant Information sheet and informed consent were embedded into the questionnaire. Participants were made aware in the participant information sheet that they could omit any questions they did not wish to answer. The only mandatory questions were those within the consent form. Ethical approval was obtained from Aston University School of Health and Life Sciences Ethics Committee (REC ID: HLS21150).

### Survey dissemination and completion of the training package

The survey was disseminated to students prior to the training using a survey link and QR code. This pre training survey was used to assess students' baseline understanding of autistic individuals and/or people with learning disabilities. This initial survey provided insight into students‘ existing knowledge, attitudes and perceptions, serving as a point of comparison for evaluating the impact of the training. The students completed Tier 1 of the Oliver McGowan Mandatory Training, which consists of two parts. Part one of the OMMT was a 90-min E-learning that all participants were required to complete independently. This e-learning module included a built-in 5-min pre- and post-questionnaire developed by NHS England, designed to assess immediate changes in knowledge and understanding related to autism and learning disabilities. Following completion of the e-learning module, students attended part two of the tier 1 training, which was delivered through a 1-h interactive webinar. This live session was co-facilitated by trained professionals and experts by experience, providing students with the opportunity to engage in reflective discussion and deepen their understanding of autism and learning disabilities through real-world perspectives. Post completion of both components of Tier 1 OMMT training, students were asked to complete a post-training survey. This allowed researchers to assess any changes in knowledge, attitudes, or perceptions compared to the pre-training responses.

### Data analysis

Data analysis involved using a mixed-methodology approach. Likert scale questions were analyzed using quantitative analysis. Likert scale data was converted to a numerical scale to facilitate statistical analysis via non-parametric methods. For agreement statements, this included; 4 = *strongly agree, 3* = *agree, 2* = *disagree, 1* = *strongly disagree*. For statements assessing students' confidence the following scale was used; *4* = *very confident, 3* = *fairly confident, 2* = *not very confident, 1* = *not confident at all*. To compare the pre-training and post training a One-way ANOVA was carried out and corrected with Levene's test (24, 25) using IBM-SPSS Statistics version 29. Statistical significance was set as *p* < 0.05.

Qualitative data were analyzed using thematic analysis following Braun and Clarke's (26) structured framework. This involved initial familiarization with the data, generating codes systematically, developing potential themes, reviewing and refining themes, and finalizing themes through collaborative discussion. Two researchers independently coded the data, discussed any discrepancies, and agreed on the final thematic structure to ensure reliability and rigor. The analysis was primarily inductive, allowing themes to emerge from participants' responses, while being informed by theoretical perspectives from Transformative Learning Theory and disability-affirmative frameworks, creating a hybrid inductive–theoretical approach. While Braun and Clarke's later (27) reflexive thematic analysis emphasizes a more reflexive approach, the 2006 framework provided a structured method appropriate for applied evaluation of OMMT learning outcomes in a higher education context.

**Figure 1:**
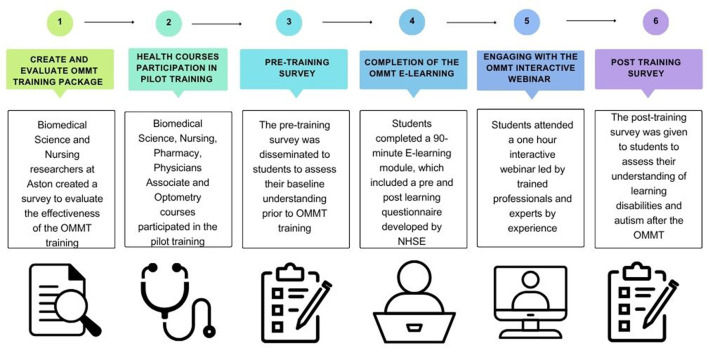
Outline of the process used to design, deliver and evaluate Tier 1 of the Oliver McGowan mandatory training. Created with Canva.com.

Figure 1 shows the methodology used to complete the evaluation of tier 1 of the Oliver McGowan Mandatory Training. A six-step process was followed, which included **(I)** creation and evaluation of the OMMT survey to evaluate the effectiveness of the training. This was created by Biomedical Science and Nursing researchers at Aston University. (II) Healthcare courses were invited to participate in the pilot training. (III) The pre-training survey was disseminated to all participating students. (IV) Students were sent a link to complete a 90-min e-learning module provided by NHS England. (V) Students attended an interactive webinar delivered by NHSE, which included experts by experience. (VI) A post-training survey was disseminated to assess students' understanding of learning disabilities and autism.

## Results

A total of 176 students completed the pre-intervention survey and 94 students completed the survey post OMMT. Results were measured using self-reported Likert-scale responses across several domains. Participants were asked a series of questions to assess their experience dealing with individuals who identified as having learning disabilities and/or autistic people. A total of 51 participants expressed having family members who have learning disabilities and/or autism. Whilst 44 respondents reported that they had worked in a care setting with individuals who have learning disabilities or autism. Twenty-nine respondents reported having friends who have learning disabilities or autism, and 12 respondents reported having work colleagues who have learning disabilities or autism. This highlighted that the majority of students had either worked with or knew of someone who identified as having a learning disability. On the contrary, 109 out of 176 participants (62%) had not heard of the LeDeR report prior to engaging with the OMMT, and pre-training only 25% of participants could correctly identify all 7 conditions (constipation, respiratory infections, heart disease, diabetes, sensory impairments, mental health problems and epilepsy) which contribute to premature mortality in individuals with learning disabilities and Autism. Finally, only 39 respondents (22%) correctly identified the estimated difference in life expectancy, in terms of years (16–20 years younger than the general population), for autistic individuals or who are differently abled.

### Quantitative analysis

Table 1 presents the statistical analysis of student responses to survey questions conducted before and after completing the Tier 1 Oliver McGowan training. Out of the 10 questions in the table, seven yielded statistically significant results with a *p*-value of < 0.0001. Post-OMMT Training students demonstrated a better understanding and awareness of mild, moderate, severe, and profound learning disabilities and autism (*p* < 0.0001). Students also reported improved confidence in verbally interacting with people who have learning disabilities and autistic people (*p* < 0.0001). In addition, students reported feeling more confident using different methods of communicating with an autistic person and an individual with learning disabilities (*p* < 0.0001). Post-OMMT training, more students were aware of the term “reasonable adjustments” for Autistic people and those with learning disabilities (*p* < 0.0001). The results indicate that the training has improved students' understanding of autism and confidence in working with and providing care for autistic and learning-disabled individuals. Importantly, when asked how important students felt it is to have an understanding and awareness of Autism and learning disabilities included in their courses, respondents reported this content as highly important prior to and post training (*p* = >0.05). Furthermore, pre- and post-training respondents identified a moderate impact on premature death for people with learning disabilities despite completion of the training, respondents were not able to identify the increased risk in reduced life expectancy for this population (*p* = 0.752).

**Table 1:** Descriptive analysis of student responses to a series of questions pre and post completion of the Tier 1 Oliver McGowan training.

**Questions**	**Pre *N* = 176, Post *N* = 94**	**Mean**	**Std. deviation**	***P*-value**
I have a good understanding and awareness of mild, moderate, severe and profound learning disabilities.	Post	3.4043	0.57397	**0.0001** ^*^
	Pre	2.9519	0.67997	
I have a good understanding and awareness of Autism.	Post	3.4891	0.50262	**0.0001** ^*^
	Pre	2.9774	0.71603	
How confident are you in verbally interacting with people that have learning disabilities.	Post	3.234	0.45022	**0.0001** ^*^
	Pre	2.7889	0.68702	
How confident are you in verbally interacting with Autistic people.	Post	3.2766	0.47302	**0.0001** ^*^
	Pre	2.7852	0.70458	
How confident are you in using different methods of communicating with an autistic person.	Post	3.1915	0.5541	**0.0001** ^*^
	Pre	2.6444	0.7563	
How confident are you in using different methods of communicating with a person with learning disabilities.	Post	3.2151	0.56817	**0.0001** ^*^
	Pre	2.6654	0.7724	
How important do you think it is to have understanding and awareness of Autism included in the curriculum for your course.	Post	3.883	0.32317	0.171
	Pre	3.8148	0.45111	
How important do you think it is to have learning disabilities included in the curriculum for your course.	Post	3.8617	0.34706	0.121
	Pre	3.777	0.51332	
Have you come across the term “reasonable adjustments” for Autistic people and those with learning disabilities?	Post	1.8617	0.34706	**0.0001** ^*^
	Pre	1.5259	0.50025	
What impact do you think coming from an ethnic minority background has on the risk of premature death for people learning disabilities?	Post	2.3936	0.55296	0.752
	Pre	2	0.057	

Values shown in bold and ^*^ denote statistically significant results (*p* < 0.05).

### Thematic analysis

**Figure 2:**
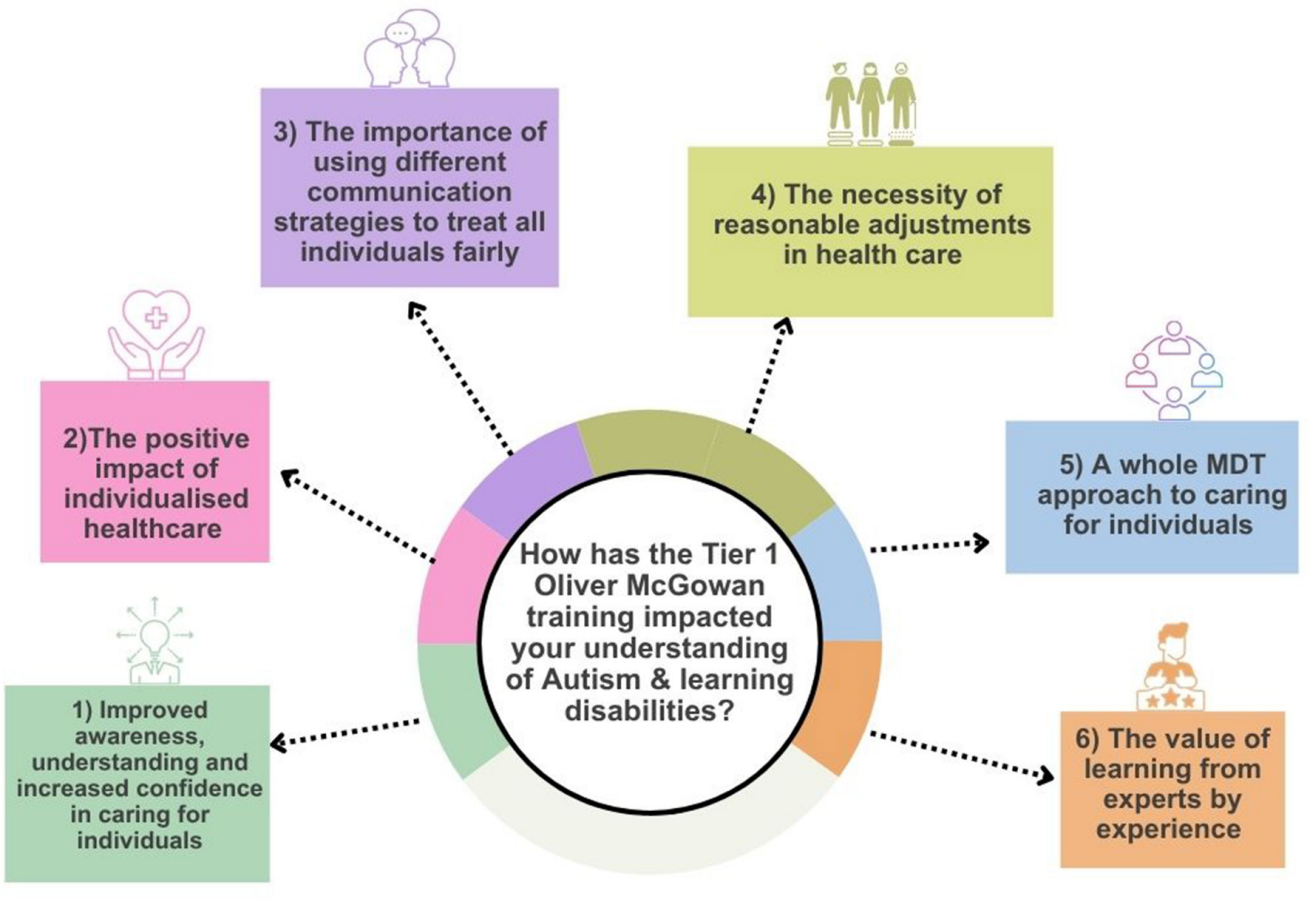
The impact of the Tier 1 Oliver McGowan training on healthcare students understanding of autism and learning disabilities. Created with Canva.com.

Free-text responses provided by students offered insight into how the Tier 1 Oliver McGowan Mandatory Training influenced not only what students learned, but how they began to think about their future professional roles. Analysis of responses across all three open-ended questions revealed six interrelated themes that reflect shifts in awareness, values, and professional identity rather than simple recall of training content (Figure 2). Many responses contributed to more than one theme, indicating that students experienced the training as an integrated learning process rather than discrete knowledge acquisition.

The Tier 1 Oliver McGowan training has had a multi-faceted impact on healthcare students understanding and approach to autism and learning disabilities. Through the thematic analysis several prominent themes have emerged that demonstrate both attitudinal and behavioral shifts in relation to providing care and working with individuals. These included:

- Improved awareness, understanding and increased confidence in caring for autistic people with learning/intellectual disabilities.
- The positive impact of individualized care.
- The importance of using different communication strategies to treat all individuals fairly.
- The necessity of reasonable adjustments in healthcare.
- A whole MDT approach to caring for individuals.
- The value of learning from experts by experience

A central theme was increased awareness, understanding, and confidence, which extended beyond factual learning to a re-evaluation of prior assumptions. Students frequently described being “*surprised” in how the expert by experience who is a university graduate had overcome delays*. Students also reported having their expectations challenged, particularly through exposure to experts by experience. This suggests that the training functioned as a catalyst for perspective change, encouraging students to question stereotypes and deficit-based assumptions about autism and learning disabilities. Rather than positioning individuals as passive recipients of care, students began to recognize competence, agency, and individuality.

Closely linked to this was the theme of individualized care, which reflected a shift away from standardized or assumption-led practice toward a more person-centered approach. Students demonstrated an emerging understanding that equitable care requires adaptation, listening, and responsiveness rather than uniform treatment. Importantly, several responses highlighted increased awareness of hidden disabilities, suggesting that students were beginning to recognize how conventional clinical practices may unintentionally exclude or disadvantage some patients.

“*I will use the training to ensure the patient care I provide is accommodating to people with special needs and will offer reasonable adjustments when needed. I understand autism and learning difficulties are not like other disabilities which are commonly physically noticed and that they may me hidden.”*

“*I feel more confident in supporting these individuals as well as trying to be adaptive and make reasonable adjustments. The most essential information I learnt is always LISTEN, without assuming and without making your own conclusions. I have also learnt that it can be condescending to talk down and to almost treat them as if they were children. I feel it has impacted me and I understand that this can be seen as rude.”*

“*It opened my eyes as to how even within people who are practicing and have already been working in clinical settings lack the understanding on how to care and approach those with autism and/or learning disabilities. It shouldn't have gotten to a stage where what happened with Oliver happened and I am glad I have had this training so that I know what I can do as a HCP in these situations. My previous job as a support worker also helped as I worked with service users who had autism and learning disabilities as well as physical disabilities and so this training assisted the knowledge I had already gained*.

Students gained a deeper understanding of the *term “reasonable adjustments,”* highlighting a shift toward inclusive care planning. One respondent stated: “*I will use the training to ensure the patient care I provide is accommodating… and will offer reasonable adjustments when needed.”* Notably, several respondents referred to previously held misconceptions especially regarding hidden disabilities and demonstrated an increased understanding and awareness that invisible cognitive or communicative differences require just as much consideration as physical impairments.

The theme of communication strategies revealed how students began to translate this awareness into practical action. Rather than viewing communication as a neutral or technical skill, students recognized it as relational and ethical, with the potential to either empower or marginalize patients. Reflections on avoiding condescension and adapting communication styles indicate growing reflexivity about professional behavior and power dynamics within healthcare encounters. One respondent stated “*I found learning about communication strategies, reasonable adjustments, and being able to recognize distress signals… enhances our understanding and awareness.”* By recognizing and responding to non-verbal cues, healthcare professionals will be able to provide more patient centered care.

The emphasis on a multidisciplinary team (MDT) approach highlights how students began to situate their learning within wider healthcare systems rather than individual practice alone. Responses indicated growing awareness that inclusive care depends on collaboration, shared responsibility, and consistent communication across services, reflecting early professional socialization into team-based care models.

Finally, the value of learning from experts by experience emerged as a key mechanism underpinning many of the other themes. Engagement with lived experience appeared to humanize abstract concepts, deepen emotional engagement, and prompt critical reflection. Rather than simply reinforcing training messages, these encounters helped students understand the real-world consequences of poor care and the importance of empathy, dignity, and advocacy. This suggests that lived experience acted as a transformative element, bridging the gap between theoretical knowledge and professional values.

Together, these themes indicate that the training supported both cognitive and affective learning, shaping how students understand their future professional responsibilities rather than merely what they know about autism and learning disabilities.

**Figure 3:**
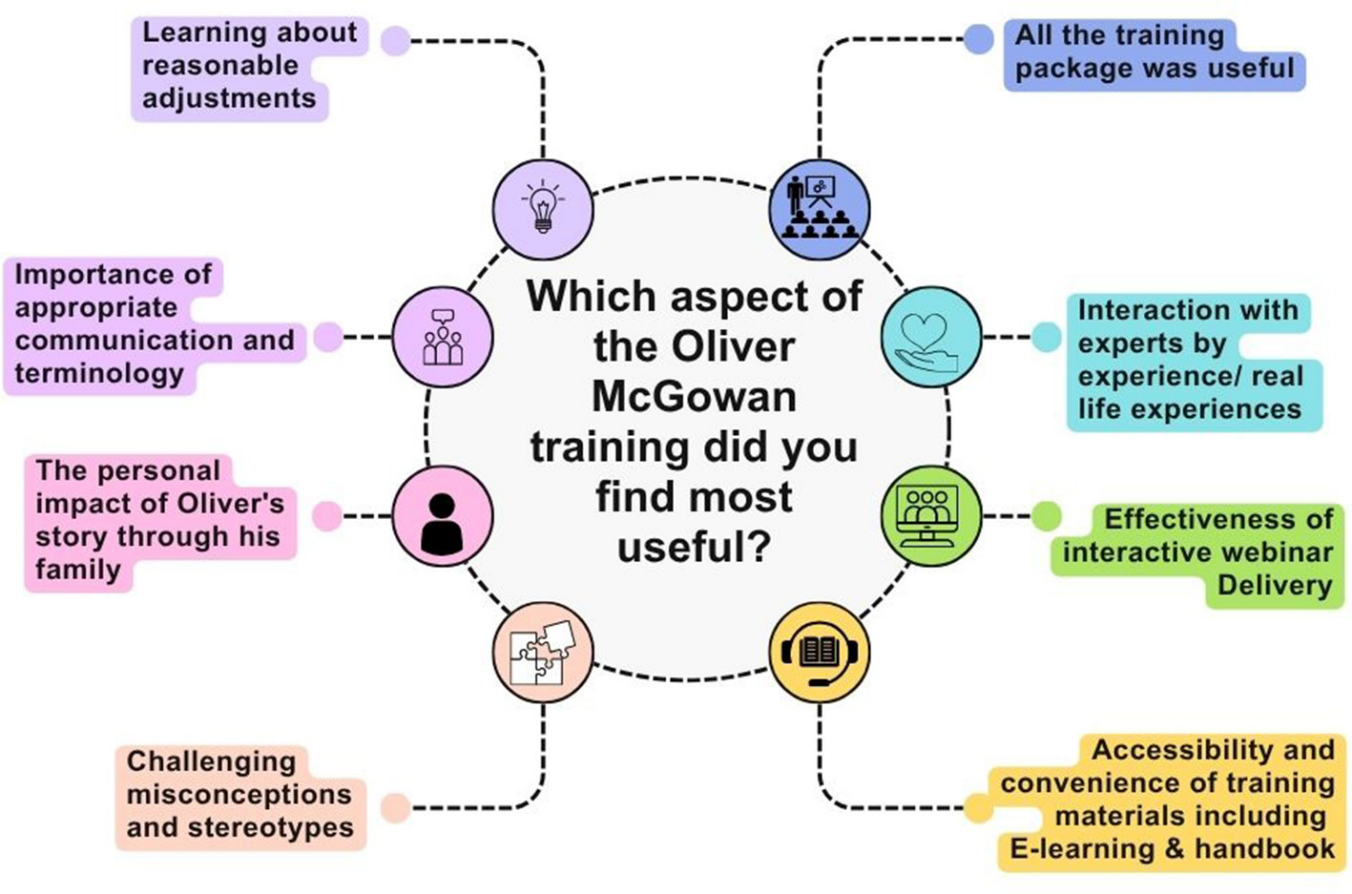
Displays the aspects of the Tier 1 Oliver McGowan training that healthcare students found most useful. Created with Canva.com.

Students were asked to comment on the aspects of the Tier 1 Oliver McGowan training they found was most useful. Several key aspects were highlighted as shown in Figure 3. Many students reported that “*the whole training package was useful*.” Healthcare students really valued the inclusion of the experts by experience and the inclusion of Olivers family's story in the webinar:

“*I really valued hearing from experts from experience, and the online session was particularly interesting, educational and insightful. Having continued access to the e-learning and handbook is beneficial, as I feel it is a useful resource that I will continue to reference going forward.”*

“*I found it useful having videos of actual individuals with autism explaining what makes things difficult for them/easy. Also found it very useful that Olivers mom was explaining the story there was a lot of detail and she was very clear in explaining.”*

*The most useful aspect of the Oliver McGowan training is its focus on real-life experiences shared by individuals with autism and learning disabilities. These personal insights help to contextualize the challenges they face, making the training relatable and impactful.”*

The strong emphasis placed on experts by experience suggests that learning was most impactful when students encountered authentic, first-hand accounts rather than abstract descriptions of need. These narratives appeared to humanize healthcare encounters and challenge pre-existing assumptions, enabling students to reframe autism and learning disability from a deficit-based perspective to one centered on capability, context, and dignity. This finding points to the importance of relational and experiential pedagogies in shaping early professional values.

Respondents also appreciated the two-part structure of the training package as it made them recognize the importance of making reasonable adjustments and communicating effectively:

“*The webinar with individuals with learning disabilities and autism was helpful as I was able to understand their experiences in healthcare and how it affects them and what reasonable adjustments may be helpful.”*

“*Learning about different communication strategies, reasonable adjustments and being able to recognize distress signals”*

“*It enables you to identify people with autism and learning difficulties better, therefore allowing you to adjust your communication style to ensure you can get all the information you need to treat the patient with the highest level of standard”*

Overall, these responses suggest that students were not only able to identify useful components of the training but also articulate how these experiences reshaped their understanding of professional responsibility, empathy, and person-centered care. The prominence of lived experience and narrative learning indicates that affective and reflective learning processes played a central role in how the training influenced students' perspectives.

### Understanding inequity in healthcare access

To further emphasize the impact of accessing healthcare, students were asked if they thought Autistic people and those with learning disabilities faced issues accessing health services. A total of 51% agreed with the statement pre training and 81% agreed post-training. In addition to lack of accessibility due to physical and systemic barriers such as transport, analysis of the free text responses revealed the reasons several recurring themes as to elucidating the barriers faced by these individuals:

*Lack of knowledge and confidence in seeking support:* Students recognized that individuals often lack the knowledge or confidence to seek appropriate support. Specific comments include, “*It's difficult for them to communicate what could be wrong,” and “having fears and anxieties about attending a care setting due to previous poor experience or sensory issues. “Appointments are booked in a way they can't fully access”* highlighting the challenges in expressing health concerns.

*Underfunded NHS services and lack of awareness:* Respondents recognized that NHS services related to autism and learning disabilities are often “*severely underfunded*,” leading to a general lack of awareness about available resources.

*Stigma attached to learning disabilities and fear of dismissal/discrimination:* The stigma attached to learning difficulties was identified as a significant barrier. Specific comments included “*People have a stigma attached to learning difficulties and some HCP may treat these patients differently*” and “*individuals may fear judgement and information may not being provided in accessible format leading to diagnostic overshadowing”* this emphasizes concerns about potential biases in receiving healthcare.

*Appointment-related challenges and sensory sensitivities:* Issues such as attending appointments, the length of appointments, and the way they are scheduled were also highlighted. Responses highlighted concerns such as, “*Appointments being booked in a way they can't fully access,” indicating logistical challenges in healthcare access” and “needing flexible appointments times (a longer session), “standard/long appointment times may result in sensory overload. There may be difficulties attending appointments due to transportation issues and issues communicating with healthcare providers.”* Students recognized that standard healthcare environments might not accommodate sensory sensitivities common amongst autistic individuals, leading to discomfort or avoidance of medical settings

*Misunderstanding symptoms and diagnostic overshadowing: students were aware of* diagnostic overshadowing; this term explains where new symptoms are attributed to existing disabilities*; “Misunderstanding of Symptoms- difficulty in expressing or recognizing symptoms may result in delayed or inadequate medical attention.”* was highlighted.

*Reliance on others to advocate*: The requirements for individuals to rely on others to access appropriate healthcare and advocate on their behalf was a recurrent theme “*They may need to rely on others, like friends or family.”*

*Communication difficulties:* there were numerous concerns around effective communication between healthcare staff, autistic individuals, and those with learning disabilities was a significant concern. Respondents highlighted: “*It's difficult for them to communicate what could be wrong. People have a stigma attached to learning difficulties and some HCP may treat these patients differently.”* Furthermore, concerns around misdiagnosis “*wouldn't be able to explain what their problem is in a way the healthcare team would understand fully, this could lead to misdiagnosis.”*

*Inadequate professional training*: another recurring theme was a fear that there was insufficient training amongst healthcare professionals in responding to the specific needs of individuals with autism and learning disabilities; “*Healthcare workers may not have enough training on how to deal with individuals with learning disabilities and often may misjudge or be unaware of how to communicate properly.”*

This analysis highlights that through the training package, healthcare students have understood the multifaceted challenges faced by autistic individuals and those with learning disabilities in accessing equitable healthcare.

## Discussion

The Tier 1 OMMT on learning disability and autism equips healthcare students with essential knowledge and practical strategies to provide inclusive, person-centered care. By fostering greater awareness, empathy, and understanding of individual needs, the training contributes directly to SDG 3 (Good Health and Wellbeing) by supporting mental health, reducing healthcare inequalities, and improving safety and outcomes for autistic people and those with learning disabilities. It also aligns with SDG 4 (Quality Education) by promoting inclusive learning environments where all students, regardless of ability, are prepared to meet diverse patient needs. Framing the training in the context of these global goals reinforces the broader significance of equipping future healthcare professionals with the skills, confidence, and attitudes needed to deliver equitable and compassionate care, while situating local educational interventions within an international agenda for health and education equity (28, 29).

Multiple studies support the inclusion of autism and learning disability training for practicing healthcare professionals (10, 15, 30–32). However, there remains a critical gap in studies focusing on healthcare students. This is the first study to evaluate the inclusion of Tier 1 OMMT into healthcare courses in higher education. Addressing this gap is essential to fostering a more inclusive and informed healthcare workforce, ultimately enhancing patient safety and outcomes for autistic people and those with learning disabilities. Legislative frameworks in England, including the Autism Act (6) and the Health and Care Act (8), mandate that healthcare providers ensure staff receive training appropriate to autism and learning disabilities, reinforcing the statutory expectation of competence in these areas. Limiting this training to professionals is insufficient; all allied health students must receive education before entering clinical practice. Without comprehensive training at undergraduate and postgraduate levels, newly qualified healthcare professionals risk perpetuating the same systemic failures that continue to disadvantage autistic people and those with learning disabilities.

## Understanding and communication

This study demonstrates that undergraduate and postgraduate healthcare students' self-perceived understanding of autism and learning disabilities increased significantly (*P* = 0.0001, Table 1). Students also reported increased confidence in working with or caring for autistic people and those with learning disabilities (*P* = 0.0001). This aligns with findings from the National Development Team for Inclusion (NDTI) on OMMT in healthcare settings, which reported improved understanding and confidence among staff. The involvement of experts by experience was consistently identified as a key strength of the training (33). Our results highlight the value of integrating such training into higher education courses rather than limiting it to qualified healthcare professionals. Incorporating OMMT into university curricula ensures the next generation of professionals is better prepared to deliver equitable care.

Students reported increased confidence in communicating with autistic people and those with learning disabilities (*P* = 0.0001); however, it is unclear whether this includes alternative methods of communication, such as British Sign Language, vocalizations, gestures, or picture-based systems. Many autistic people and individuals with learning disabilities use few or no words, and their ability to communicate verbally can vary, for example, if they are overstimulated or dysregulated (50). Lack of familiarity with these communication methods has led to dismissal or neglect of patients, as illustrated by the case of Oliver McGowan (5, 10). Healthcare professionals must therefore become competent in alternative communication strategies to provide equitable, person-centered care. This remains an important area for ongoing development in OMMT.

## Shaping future professional perspectives through training

The six themes identified in the evaluation; improved awareness and confidence, the positive impact of individualized care, the importance of varied communication strategies, the necessity of reasonable adjustments, a whole multidisciplinary team approach, and the value of learning from experts by experience offer more than a summary of training content. Viewed through the lens of Transformative Learning Theory (34), these themes suggest genuine shifts in students' perspectives and professional socialization. Students did not merely recall factual content; they reported a deeper understanding of the complexity of caring for autistic individuals and people with learning disabilities, reflecting a re-evaluation of prior assumptions about patient care.

The prominence of learning from experts by experience indicates that direct engagement with lived experience is a powerful driver of reflection and attitudinal change. Hearing first-hand accounts appeared to humanize the conditions and challenges faced by patients, reinforcing person-centered care principles and helping students internalize the importance of empathy, flexibility, and advocacy. This aligns with literature highlighting that exposure to lived experience can foster reflective practice, challenge stereotypes, and bridge the gap between theoretical knowledge and clinical application (16, 17, 35).

Similarly, the emphasis on communication strategies, reasonable adjustments, and individualized care suggests that students began to recognize the nuanced ways systemic practices and professional norms can either support or hinder equitable care. The responses indicate an emerging awareness of their own role as future healthcare professionals in creating inclusive environments and how interprofessional collaboration enhances patient outcomes. In other words, the training seems to have facilitated both cognitive and affective learning, encouraging students to reconsider not just “how” to care, but “why” these practices matter.

Observed increases in students' understanding, confidence, and appreciation of lived experience align with Transformative Learning Theory, suggesting genuine shifts in perspectives rather than rote compliance with training objectives. Students not only demonstrated improved knowledge of autism and learning disabilities but also reported greater confidence in applying communication strategies and reasonable adjustments in practice. The incorporation of experiential learning, interprofessional collaboration, and engagement with experts by experience appears to have encouraged critical reflection on prior assumptions and fostered a deeper awareness of systemic barriers faced by autistic people and people with learning disabilities. This indicates that the OMMT may facilitate meaningful attitudinal and behavioral change, equipping students to provide more inclusive, person-centered care and preparing them to challenge ableist assumptions embedded in traditional healthcare practice.

The findings of this study align closely with the conclusions of a recent mixed-methods systematic review by Franklin et al. (20), which evaluated educational interventions for health professionals in autism and learning disability. Their review identified consistent improvements in knowledge, confidence, skills, and attitudes following training, but also highlighted substantial heterogeneity in intervention design, outcome measures, and study quality. Similar to the literature reviewed by Franklin et al., the present study demonstrates positive self-reported gains in understanding and confidence following training, while also reflecting common methodological challenges, including reliance on pre–post designs and bespoke evaluation tools.

Importantly, Franklin et al. (20) identified limited involvement of people with lived experience in the design and delivery of training as a key gap in existing educational interventions. In contrast, Tier 1 Oliver McGowan Mandatory Training is explicitly co-produced and co-delivered with experts by experience, a feature that emerged as a central strength in the current evaluation. Students consistently identified lived experience input as the most impactful element of the training, suggesting that this component may be particularly influential in facilitating reflective and perspective-based learning, consistent with principles of Transformative Learning Theory.

The review also noted a lack of clarity regarding optimal delivery modes, duration, and integration of training within professional education pathways, particularly for adult-focused and pre-registration learners. By evaluating OMMT within undergraduate and postgraduate healthcare programmes, this study contributes novel evidence addressing this gap and demonstrates the feasibility and perceived value of embedding mandatory, nationally standardized training within higher education curricula. In doing so, it responds directly to Franklin et al.'s (20) call for earlier, more systematic integration of autism and learning disability education into professional training pathways. Together, these findings reinforce the importance of combining co-produced training models with robust, theory-informed evaluation approaches to strengthen the evidence base for autism and learning disability education in healthcare.

While the findings demonstrate statistically and thematically that Tier 1 OMMT enhances students' understanding, confidence, and appreciation of lived experience, it is important to note that these results reflect students' self-perceptions rather than objective measures of clinical behavior. The immediate educational impacts, including increased awareness of communication strategies, reasonable adjustments, and person-centered care, represent early shifts in professional perspectives. Potential effects on future professional practice, such as improved patient outcomes or changes in clinical behavior, remain to be determined and may require longitudinal follow-up or assessment during clinical placements. This distinction reinforces the interpretation of the study as capturing attitudinal and cognitive changes that are foundational for safe, inclusive practice, while avoiding overgeneralisation regarding direct behavioral outcomes.

## Secondary conditions contributing to poor care

The Learning from Lives and Deaths of People with a Learning Disability and Autistic People (LeDeR) programme, funded by NHS England, aims to improve services by reviewing care and highlighting inequalities (36). LeDeR reviews show that autistic people and people with learning disabilities die earlier than the general population and often do not receive the same quality of care (55). Only 25% of students correctly identified the seven conditions that contribute to premature mortality in these populations (constipation, respiratory infections, heart disease, diabetes, sensory impairments, mental health problems, and epilepsy). For example, constipation is common and preventable, yet 40% of individuals have been prescribed laxatives, and 13% died from complications such as bowel obstruction, highlighting the need for training to recognize and manage these risks. Although Tier 1 OMMT does not cover these clinical conditions in depth, Tier 2 training includes LeDeR content.

Qualitative research with learning disability psychiatry multidisciplinary teams highlights that poor healthcare quality for people with learning disabilities is driven not only by individual knowledge gaps, but by systemic failures within mainstream services, including diagnostic overshadowing, inadequate capacity assessments, poor use of health passports, and limited cross-service collaboration (3, 48). These findings closely align with the themes identified in this study, particularly students' increased awareness of communication needs, reasonable adjustments, and the importance of multidisciplinary working.

Students' reflections demonstrate an emerging understanding of how structural barriers and professional practices can contribute to inequitable care, echoing concerns raised by specialist MDTs. This suggests that Tier 1 OMMT supports early professional socialization by introducing system-level perspectives before students enter full clinical practice. By emphasizing person-centered care, communication, and shared responsibility across disciplines, the training mirrors the collaborative approaches identified as essential for improving healthcare quality.

Although the focus differs, parallels can be drawn with research on antibiotic prescribing in healthcare. Bashir et al. (37) identified critical points in the antibiotic prescribing pathway in a children's hospital, highlighting a disconnect between prescribers' knowledge and their behaviors. Despite awareness of antimicrobial resistance, many prescribers lacked confidence to adjust therapy appropriately, and training alone was unlikely to change practice without additional structured support. These findings resonate with our evaluation of Tier 1 OMMT, emphasizing that knowledge acquisition is necessary but insufficient for genuine behavior change. For healthcare students, experiential learning, engagement with experts by experience, and reflection on person-centered practice appear essential to translate understanding of autism and learning disabilities into professional attitudes and behaviors. Together, these examples reinforce the need for education that combines theoretical knowledge with practical, reflective, and co-produced learning to prepare future healthcare professionals for complex clinical decision-making.

## Interprofessional education and interdisciplinary competencies

A key pedagogical challenge is cultivating interdisciplinary competencies when academic staff are grounded in distinct disciplinary frameworks. OMMT addresses this by providing an inherently interdisciplinary training environment. Delivered to students across five healthcare courses, the training leveraged interprofessional education (IPE) principles, allowing students to learn with, from, and about each other. Facilitators from different disciplines collaborated to deliver OMMT, supported by standardized preparation and live webinars. IPE equips students with teamwork and communication skills necessary for effective multidisciplinary practice, improving patient outcomes and reducing professional silos (14, 38, 43). The OMMT also emphasizes person-centered care, encouraging partnership with patients and families, respect for autonomy, and compassionate practice.

## Policy relevance and training rollout

Training in autism and learning disabilities is a policy priority for NHS England. Over one million people have completed Tier 1 e-learning, and OMMT is now the government's preferred training programme. The 2025 Code of Practice (39) sets training standards and co-production principles. Tier 2 rollout will support the development of a robust, capable workforce. Without appropriate training, healthcare professionals risk misunderstanding communication needs, creating anxiety and distress, and perpetuating health disparities (40).

## Recommendations

From survey responses, we recommend:

- Introduce training in Year 1 and reinforce throughout the curriculum.
- Embed content in topics such as legislation, ethics, communication, and safeguarding.
- Include people with lived experience to promote authentic understanding.
- Ensure co-production in all educational materials.
- Incorporate IPE to illustrate collaborative approaches to care.
- Facilitate reflection to embed knowledge, attitudes, and behaviors.

Training healthcare students in autism and learning disabilities is essential for delivering safe, legal, ethical, and compassionate care, equipping the workforce to reduce inequalities and treat patients with dignity and respect (41).

## Limitations and future work

This is the first study evaluating OMMT in a HEI context, delivered to healthcare students at Aston University. Limitations include single-institution data, absence of medical and audiology students, lower post-training response rates, and short-term pre/post surveys without longitudinal follow-up. It is notable that no explicitly negative or ambivalent responses were captured in the post-training survey. While this may reflect genuinely positive engagement, the attrition rate (47%) may also suggest a degree of ambivalence or disengagement among non-responders. This reinforces the importance of interpreting the results with caution while also highlighting the challenge of capturing the full range of student perspectives in higher education, where survey participation is often voluntary, unincentivized, and subject to timing constraints such as exams or placement commitments.

While a reduction in responses was observed between the pre-training (*n* = 176) and post-training (*n* = 94) surveys, this should be interpreted within the context of higher education research practice. Although completion of the Tier 1 Oliver McGowan Mandatory Training was mandatory within the curriculum, participation in the pre- and post-evaluation surveys was entirely voluntary. As such, students were under no obligation to complete the post-training survey, which inevitably influenced response rates. Lower response rates in student survey research are well-documented, particularly where no incentives are offered (14, 42). No monetary or academic incentives were associated with survey completion in this study.

It is also important to note that the OMMT package itself includes an in-built feedback survey. It is therefore likely that some students perceived this as fulfilling the requirement to provide feedback and may not have recognized the need to complete an additional institutional evaluation survey, further contributing to reduced post-training response rates. Unfortunately, responses from this in-built survey were not shared with the HEI.

Furthermore, to avoid any perception of coercion, survey invitations and reminders were distributed by programme staff who were not involved in the delivery and evaluation of the training, in line with ethical committee guidance. While necessary to satisfy ethical requirements, this approach is recognized to reduce engagement, as students are typically more responsive to communication from staff directly involved in their teaching. The timing of the post-training survey also likely contributed to attrition. By the time the follow-up survey was disseminated, some students had entered examination periods or commenced clinical placements, limiting their availability and capacity to engage with non-compulsory research activities.

Taken together, these factors highlight that the observed attrition is largely attributable to structural, ethical, and practical constraints within a higher education context, rather than a lack of engagement with the training itself. Where feasible, pre- and post-training responses were paired for individual students, enabling within-subject comparisons to provide a robust assessment of changes in knowledge, confidence, and preparedness. Furthermore, the authors have conducted statistical analysis on pre and post cohorts, in a bid to determine differences in cohort characteristics. We conclude, after comparing age, gender, year of study and programme of study, the pre and post cohorts did not exhibit statistically significant differences (*P* = >0.05). Thereby, we report that despite the attrition observed the data is comparable between pre and post sets, as well as a student sample, consistent with Aston University.

While the bespoke survey was closely aligned with the Tier 1 OMMT learning outcomes, it has not undergone formal psychometric validation. Future work could assess its reliability and construct validity, particularly to support replication across different higher education contexts. This study employed a quasi-experimental pre–post design without a control group, which is well-suited to evaluating the real-world implementation of a nationally mandated training programme within a higher education context. The significant improvements observed across students' self-reported knowledge, confidence, and understanding of reasonable adjustments provide strong initial evidence of the value of Tier 1 OMMT for healthcare students. While causal inferences should be interpreted with appropriate caution, as is standard in educational implementation research, the consistency of findings across quantitative and qualitative data strengthens confidence that the observed changes are meaningfully associated with the training. These results offer an important foundation for future research, including longitudinal or comparative studies, and support the continued integration of mandatory, co-produced autism and learning disability training within healthcare curricula.

Despite these constraints, the post-training sample remained representative of multiple healthcare programmes and levels of study, allowing meaningful exploration of student perceptions and learning outcomes. Nevertheless, as with all voluntary self-report studies, it is possible that students who completed the post-training survey were more engaged with the training, and therefore the observed improvements may overestimate effects across the full cohort. This limitation is acknowledged, and future research would benefit from more paired longitudinal designs, improved follow-up strategies, or ethically appropriate incentives to enhance response rates and strengthen the evaluation of training effectiveness. Future research should also expand to multiple HEIs, incorporate longitudinal designs, and evaluate clinical translation.

While Tier 1 OMMT demonstrates clear educational value, its sustainability within higher education institutions may be influenced by wider structural and financial pressures. Ongoing funding constraints in both the NHS and higher education, alongside continued organizational restructuring, may affect institutions' capacity to support consistent delivery and evaluation at scale. Although OMMT is mandated under the 8, implementation within university curricula relies on local resources, staff capacity, and effective collaboration between universities and healthcare partners. Without sustained policy commitment and secure resourcing, there is a risk of variability in delivery that could undermine the long-term integration and impact of the training in preparing future healthcare professionals.

## Conclusion

Tier 1 OMMT improves healthcare students' understanding, confidence, and preparedness in supporting autistic people and those with learning disabilities. Key outcomes include enhanced communication, appreciation of lived experience, and recognition of individualized care needs. The training aligns with policy imperatives, promoting equitable, person-centered, and compassionate healthcare. Embedding autism and learning disability education across HEIs is essential to prepare a competent, inclusive, and ethically responsible future workforce.

## Data Availability

The original contributions presented in the study are included in the article/supplementary material, further inquiries can be directed to the corresponding author.
